# CpG methylation patterns of human mitochondrial DNA

**DOI:** 10.1038/srep23421

**Published:** 2016-03-21

**Authors:** Baojing Liu, Qingqing Du, Lu Chen, Guangping Fu, Shujin Li, Lihong Fu, Xiaojing Zhang, Chunling Ma, Cong Bin

**Affiliations:** 1 Hebei Key Laboratory of Forensic Medicine, Department of Forensic Medicine, Hebei Medical University, No. 361 Zhong Shan Road, Shijiazhuang, Hebei 050017, People’s Republic of China

## Abstract

The epigenetic modification of mitochondrial DNA (mtDNA) is still in controversy. To clarify this point, we applied the gold standard method for DNA methylation, bisulfite pyrosequencing, to examine human mtDNA methylation status. Before bisulfite conversion, BamHI was used to digest DNA to open the loop of mtDNA. The results demonstrated that the linear mtDNA had significantly higher bisulfite conversion efficiency compared with circular mtDNA. Furthermore, the methylation values obtained from linear mtDNA were significantly lower than that of circular mtDNA, which was verified by SEQUENOM MassARRAY. The above impacts of circular structure were also observed in lung DNA samples but not in saliva DNA samples. Mitochondrial genome methylation of blood samples and saliva samples from 14 unrelated individuals was detected. The detected regions covered 83 CpG sites across mtDNA including D-loop, 12 S rRNA, 16 S rRNA, ND1, COXI, ND3, ND4, ND5, CYTB. We found that the average methylation levels of nine regions were all less than 2% for both sample types. In conclusion, our findings firstly show that the circular structure of mtDNA affects bisulfite conversion efficiency, which leads to overestimation of mtDNA methylation values. CpG methylation in human mtDNA is a very rare event at most DNA regions.

Whether mammal mitochondrial DNA (mtDNA) is methylated as nuclear DNA (nDNA), is still a controversial question with different opinions. In our recent study about twin and DNA methylation, we found that DNA methylation level at 6 CpG sites in mtDNA D-loop region in human blood samples averaged 2% to 34%, while nearly 0% in saliva samples. Furthermore, it showed a significant difference within monozygotic (MZ) twin pairs and could distinguish 31.93% of the MZ twin pairs (unpublished data). Therefore, the mtDNA methylation was thought to be a potential marker for discriminating two individuals of identical twin pairs. However, unexpectedly, our following study showed that there was almost no methylation found on other detected regions of mtDNA. These unexpected results prompt us to find the answers behind it.

Researches of mtDNA epigenetics are not as common as nDNA. The earliest study reported on mtDNA methylation appeared about 40 years ago. In mouse and hamster cell lines, mtDNA was found to be significantly less methylated than nDNA with respect to 5-methylcytosine (5 mC), and the level of 5 mC in mtDNA as compared with that in nuclear DNA was estimated as one-fourth to one-fourteenth in various cell lines^1^. Other studies on cows and rats showed that the 5 mC content in mtDNA (3.03 and 2.0 mole %, respectively) is twice as much as in corresponding nDNA, and the animals display species specificity with respect to the 5 mC content in the mtDNA^2^,^3^,^4^. On the other hand, 5 mC has not been detected in mtDNA from *Paramecium aurelia*^5^, HeLa cells and *Xenopus laevis*^6^. The internal cytosine residue in the sequence CCGG of mtDNA from different strains of yeast, *Neurospora crassa*, rat and calf, was also found to be unmethylated^7^.

All of the above studies done in 1970 s used the methods to measure the 5 mC content in mtDNA or nDNA, which have been replaced by several more sensitive techniques. For example, using the nearest neighbor analysis and restriction cleavage techniques to examine the methylation pattern of mouse mtDNA, Pollack *et al*.^8^ found that methylated cytosine appeared exclusively in the dinucleotide sequence CpG at an extremely low extent of 3 to 5%, and they pointed that such low levels of 5 mC could not have been detected by most of the procedures used previously. Applying restriction cleavage with enzymes *Msp*I and *Hpa*II to estimate the extent of methylation in human fibroblast mtDNA, Shmookler *et al*.^9^ found that approximately 2~5% of mtDNA molecules were essentially fully methylated at -CCGG- sites, while the remainder are fully unmethylated.

Nowadays, several innovative approaches for measuring DNA methylation have been established based on different techniques. For instance, liquid chromatography-electrospray ionization tandem mass spectrometry (LC-ESI-MS/MS) offers a fundamental tool for global quantitation of DNA methylation^10^. By applying this method, human global mtDNA was detected to undergo methylation^11^. ELISA was also used to verify the existence of 5 mC in mtDNA^12^,^13^. Gene specific methylation approaches are mainly based on affinity enrichment, restriction enzymes digestion or bisulfite conversion^10^. Using affinity-based methods such as 5 mC and 5 hmC immunoprecipitation, both 5 mC and 5 hmC modification were found to be present in D-loop region of mtDNA in human and mouse blood and cultured cells^14^,^15^. Although affinity-based methods are fast and efficient, they cannot provide the methylation status of individual CpG dinucleotides. Bisulfite conversion coupled with sequencing is the best way to evaluate the methylation status with single base resolution. Recently, several studies have evaluated the methylation of mtDNA by using bisulfite sequencing or pyrosequencing or specific PCR, most of the studies reported that mtDNA methylation could be detected within a level of 2% to 18% in D-loop region or 12 s rRNA or 16 s rRNA in human peripheral blood, cord blood, placenta, and mouse brain, liver, and testes^16^,^17^,^18^,^19^. On the contrary, two studies showed that methylation of mtDNA was a rare event or absent in the detected specific regions in human cell lines and primary cells^20^,^21^.

These discordant results let us to discover the real reasons for such a dispute and to map human mitochondrial genome methylation. Although a recent study by Ghosh et al described a comprehensive map of methyl cytosines across human mitochondrial genome by analyzing 39 MeDIP datasets and a bisulfite sequencing dataset, it did not provide any information on the sites or frequency of CpG methylation of the targets^22^. Bisulfite pyrosequencing technology is widely recognized as the gold standard of DNA methylation analysis, which can get precise quantification of each CpG site, and has higher accuracy through internal quality control monitoring bisulfite conversion efficiency^23^,^24^. Therefore, this study applied bisulfite pyrosequencing technology to map the methylation profile of human mtDNA.

## Results and Discussion

### Circular structure of mtDNA affects bisulfite conversion

**Figure 1:**
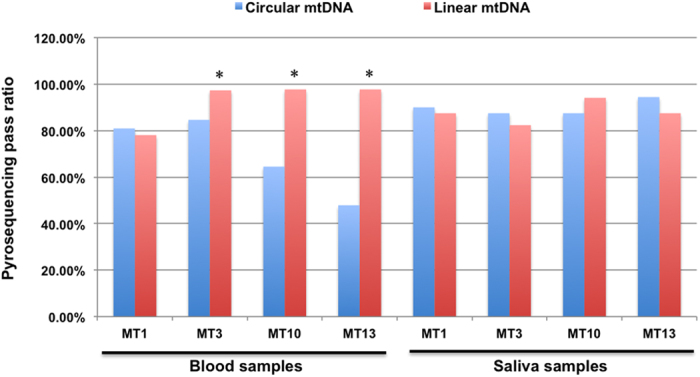
The circular structure of mtDNA affects the pyrosequencing pass ratio in blood samples but not in saliva samples. In blood samples, pyrosequencing pass ratio in linear mtDNA group was significantly higher than that of circular mtDNA group. In saliva samples, there were no significant differences between two groups. ^*^P < 0.01 *vs* linear mtDNA group.

To observe whether the ring structure of mtDNA affects the bisulfite conversion efficiency and pyrosequencing results, genomic DNA was treated with BamHI before bisulfite conversion. Four sequences MT1, MT3, MT10, MT13 located in D-loop, 12 s rRNA, ND1, and COX I (Table 1) were chosen to analyze the loop structure effect on bisulfite conversion. Through the detection of 154 blood samples, we found the sequencing success rate of four sequences MT1, MT3, MT10, MT13 in linear mtDNA group were 78.07%, 97.32%, 97.73%, 90.26%, while in circular mtDNA group were 80.95%, 84.62%, 64.52%, 47.89%, respectively. According to statistical analysis, except MT1 (*P* = 0.621), the success rate of the remaining three sequences between linear mtDNA group and circular mtDNA group had significant differences, and the success rate of circular mtDNA group were significantly lower than that of linear mtDNA group (*P* = 0.002 for MT3, *P* < 0.001for MT10 and MT13, Fig. 1).

The main reasons leading to failure in bisulfite pyrosequencing include incomplete bisulfite conversion that can be evaluated by internal quality control during pyrosequencing, low signal-to-noise ratio or signal loss caused by insufficient PCR products, and uncertain reference sequence pattern due to stochastic effect. Through further analyzing the causes for sequencing failure, we found that in the circular mtDNA group, the proportion of the unpassed inner quality control in all of the four sequences except MT3, were more than 80%. While in the linear mtDNA group, the main reason for sequencing failure was low signal-to-noise ratio or signal loss (Table 2), which were usually related to the experiment operation or caused by some stochastic effect. Failure of inner quality control was shown as higher C/T ratio than threshold in non-CpG cytosine, indicating that bisulfite conversion was not complete. Therefore, the above results showed that the circular structure of mtDNA in blood samples obviously affected the bisulfite conversion process, and the conversion efficiency was significantly decreased compared with that of linear mtDNA treated by BamHI.

Methylation modification in mtDNA is still in dispute, although numerous innovative approaches for measuring DNA methylation have been used to measure it. This study showed that the circular structure of mtDNA was a possible reason for the discordant results obtained by using bisulfite-based methods. To our knowledge, it is the first time to compare the methylation analysis differences between primary mtDNA with loop structure and linear mtDNA treated with restriction endonuclease. We found that the circular structure of mtDNA hampered the bisulfite conversion process to some extent. This is an important finding that has previously been neglected.

### Overestimation of CpG methylation values due to uncompleted bisulfite conversion

**Figure 2:**
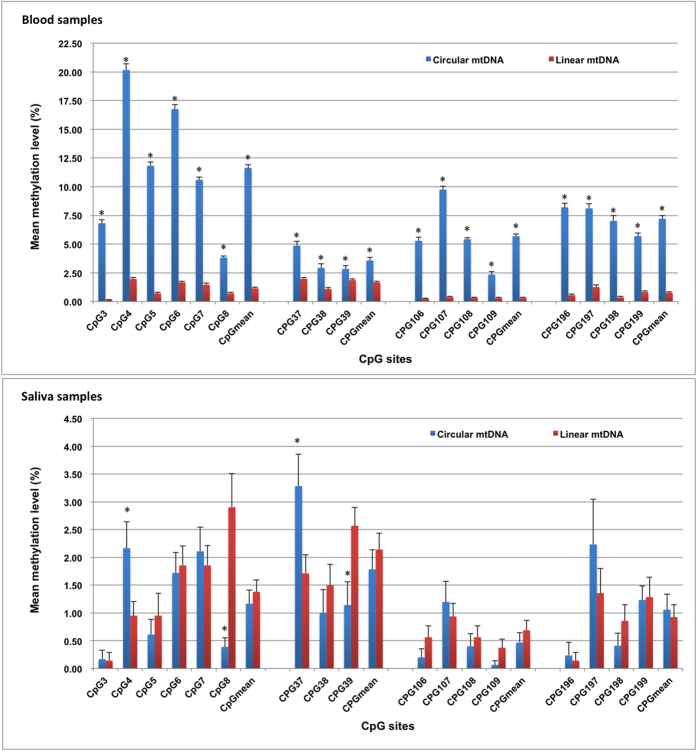
Comparison of mean methylation level between linear mtDNA group and circular mtDNA group. Data are represented as mean ± SE, ^*^P < 0.001 *vs* linear mtDNA group. For blood samples, the mean methylation values in samples of circular mtDNA group were significantly higher than that of linear mtDNA group at all of the detected CpG sites. However, for saliva samples, significant differences were only observed at four CpG sites, and two of them are higher in linear mtDNA group, the other two are just opposite.

Although the sequencing success rate of the circular mtDNA group in blood samples was greatly lower, more than 47% of the samples still succeeded in pyrosequencing. Therefore, we compared the methylation quantification values between the two groups. The results showed that the average methylation levels of MT1, MT3, MT10, MT13 were 11.65% ± 0.29%, 3.58% ± 0.27%, 5.68% ± 0.20%, 7.21% ± 0.27% in circular mtDNA group, while they were 1.16% ± 0.09%, 1.68% ± 0.10%, 0.32% ± 0.05%, 0.78% ± 0.09%, respectively in linear mtDNA group. Wilcoxon signed-rank test showed that significant differences exist between the two groups (*P* < 0.001 for all of the CpG sites), and the methylation degree in linear mtDNA group was significantly lower than that of circular mtDNA group (Fig. 2). For the same samples tested by both methods and for which methylation values were obtained, paired statistic test was performed and the results demonstrated that the D values within two methods of all the 17 CpG sites except CpG39 were statistically significant (Table 3), which further confirmed that the methylation values obtained from the circular mtDNA were significantly higher than that of the linear ones.

Therefore, if we apply bisulfite-based methods to analyze mtDNA methylation without opening loop before conversion, the results are unreliable. Our findings remind us that even pyrosequencing is performed without opening-loop treatment, the methylation values tend to be overestimated. Bisulfite-based sequencing has been used to examine cytosine modification for 20 years and remains the “gold standard” for DNA methylation analysis^25^. After bisulfite treatment, unmodified cytosine can be converted to uracil, but modified 5 mC and 5 hmC cannot be converted. Methylation modification is then changed to T/C variation, which can be detected and the methylation levels can be quantified by calculating the C/T ratio. So, if this process is not efficient, some unmodified cytosine will be not converted and remain as cytosine. As a result, C/T ratio will be increased and the methylation values will be overestimated. Some studies using bisulfite pyrosequencing to measure the methylation level of mtDNA obtained higher methylation values than ours. For example, Byun *et al*.^16^ measured methylation by bisulfite pyrosequencing in three mtDNA regions in human peripheral blood samples. They reported that the mean methylation level was 5.06% for 12 s rRNA and 2.38% for D-loop region, while ours are 1.68% for 12 s rRNA and 1.16% for D-loop region in the same kind of sample. Besides, detected by bisulfite-based methods, mean methylation degree has been reported to be 4.0% in D-loop region in human cord blood^18^, 3.7% in human placenta^18^, and 2 ~ 9% in mouse brain, liver and testes^17^. And for 12 s rRNA, the methylation value has been reported to be 11.7% in human cord blood^18^, 9.5% in placenta^18^. These methylation values might be overestimated due to incomplete bisulfite conversion.

We noticed that the study conducted by Hong *et al*.^20^ digested DNA with HindIII before bisulfite conversion. HindIII specifically recognizes AAGCTT and cuts between A/A. Three HindIII restriction sites exist in mtDNA. Therefore mtDNA will be cut into linear DNA after HindIII digestion. They then used bisulfite sequencing to detect methylation level of 12 Sr RNA, 16 S rRNA, COII and ATP6 in mitochondrial DNA in human blood cells and HCT116 cell line. The results showed that the methylation degree of these four regions were no more than 0.66%, and confirmed the absence of CpG methylation in human mtDNA, which was consistent with our studies. Therefore, we can conclude clearly that the loop structure of mtDNA must be opened before bisulfite treatment if using any bisulfite-based methods to measure mtDNA methylation, at least for blood samples, or the methylation values will be overestimated. It has been reported that bisulfite treatment might result in the fragmentation of DNA. The conditions necessary for complete conversion, such as long incubation times, elevated temperature, and high bisulfite concentration, can lead to the degradation of the incubated DNA^26^. However, since mtDNA is more stable and more anti-degraded than nuclear DNA due to the circular structure, we speculate that the intactness of mtDNA conformation might be reserved more or less. It should be confirmed by further study.

### Validation by SEQUENOM EpiTYPER MassARRAY

To validate the results obtained by pyrosequencing, we reanalyzed the mtDNA methylation status using SEQUENOM EpiTYPER MassARRAY. SEQUENOM EpiTYPER MassARRAY assay is one of the first truly high-throughput and quantitative methods to analyze DNA methylation. EpiTYPER was first introduced by Ehrich and colleagues^27^. This method to analyze bisulfite converted DNA is based on base specific cleavage of nucleotides and subsequent analysis of fragments by MALDI-TOF MS. Methylation values are given for individual CpG sites or multiple CpG sites are analysed together as CpG units depending on the cleavage pattern. The reproducibility of the methodology is remarkably consistent and the methylation values highly accurate^27^,^28^. According to the results, we found that the average methylation values of circular mtDNA without treatment of BamHI were from 3.43% to 5.93% for CpG106~CpG109, 2.79% to 27.79% for CpG225~CpG243, 1.21% to 12.57% for CpG360~CpG371, while that of linear mtDNA treated with BamHI before bisulfite conversion were from 1.07% to 3.07% for CpG106~CpG109, 1.86% to 6.86% for CpG225~CpG243, 0.57% to 2.29% for CpG360~CpG371, which were significantly reduced (Table 4). These results further confirm the pyrosequencing results, indicating that the methylation values might be overestimated under the condition of circular mtDNA without cleavage before bisulfite conversion. For the same CpG sites, the methylation values obtained by EpiTYPER were little higher than that of pyrosequencing, e,g, 1.07% to 3.07% *vs* 0.25% to 0.33% for CpG106~CpG109. It was reported that fully methylated or completely unmethylated samples show consistently a small decrease or increase in methylation, respectively^28^. Still, the sensitivity of the EpiTYPER MassARRY is 5%^28^. Therefore, we can consider that there was no methylation occurred at CpG106~CpG109 sites.

### mtDNA methylation status in saliva samples and tissue samples

Intriguingly, such effects of loop structure for bisulfite conversion efficiency were not observed in saliva samples. We detected 14 to 24 saliva samples. The sequencing success rate of MT1, MT3, MT10, MT13 in linear mtDNA group were 87.50%, 82.35%, 94.12%, and 87.50%, while that in circular mtDNA group were 90.00%, 87.50%, 87.50%, 94.44%, respectively. There was no significant difference between two groups for any of the four sequences (Fig. 1). And the pyrosequencing success rate of circular mtDNA group in saliva samples was significantly higher than that in blood samples. Even for the circular mtDNA in saliva samples that was unable to be pyroseqenced, none were caused by incomplete bisulfite conversion (Table 2). The above results showed that the circular structure of mtDNA has little effect on bisulfite conversion for saliva samples.

For saliva samples, the average methylation values of four sequences were 1.17% ± 0.25%, 1.79% ± 0.35%, 0.47% ± 0.17%, 1.06% ± 0.28% in circular mtDNA group, and 1.38% ± 0.21%, 2.14% ± 0.29%, 0.69% ± 0.18%, 0.93% ± 0.22% in linear mtDNA group, respectively. Wilcoxon signed-rank test results showed that no significant differences exist between the two groups (Fig. 2). For the same samples measured by both methods and for methylation data was obtained, paired comparison was conducted and no statistical difference was found in most of the CpG sites except for CpG8, CpG39, CpG109 sites (Table 3). These results further confirmed that the circular structure of mtDNA in saliva samples has little effect on bisulfite conversion and the subsequent methylation quantification by pyrosequencing. From these results, we understood our previous study that the methylation level of 6 CpG sites in D-loop regions is 2~34% in blood samples but nearly 0% in saliva samples. Because we didn’t perform opening-loop treatment before bisulfite conversion, the blood samples underwent an incomplete conversion while the saliva samples were not affected.

The inconsistency between blood samples and saliva samples prompt us to study other tissues. We collected 7 lung tissues from autopsies of forensic cases. The death causes of these 7 individuals were not related with lung diseases. According to the results, among the 6 CpG sites we detected, the methylation values of CpG4 and CpG5 is significantly higher in mtDNA without BamHI treatment than with BamHI treatment. Although no statistical significance was found in other 4 CpG sites, the CpG mean methylation values of the 6 CpG sites showed obvious difference between loop mtDNA group and linear mtDNA group (Table 5). The methylation value in loop mtDNA group was higher than that of linear group. These results further verified that the loop structure of mtDNA might affect efficient bisulfite conversion.

However, why saliva samples showed different results? Firstly, the saliva DNA amount we used to perform bisulfite conversion was 500 ng, while that of blood and tissue samples was 1000 ng. The excess concentration of DNA in the reaction may interfere with the ratio of bisulfite to DNA. Secondly, we compared the DNA extraction methods of blood, tissues, and saliva samples. We used QIAamp DNA kit to extract DNA from blood and tissue samples, while for saliva samples, silica and guanidinium isothiocyanate method was performed. We noticed that the pH of TE buffer used to purify blood and tissue DNA was 9.0, while that used for saliva cell DNA was 8.0. In the first step of bisulfite conversion, sulfonation, bisulfite is added to cytosine and the reaction is controlled by pH. Low pH induces formation of cytosine sulfonate while high pH reverses the reaction, with the recommended pH being around 5 to keep the process favouring efficient conversion of cytosine to uracil^29^. Therefore, relatively lower pH of saliva DNA might be one of the reasons leading to more efficient bisulfite conversion than blood and tissue samples.

For efficient bisulfite conversion, the initial denaturation step is the most critical step as deamination of cytosine to uracil requires DNA to be single-stranded^29^. Incomplete denaturation or reannealing of DNA can result in double-stranded DNA (dsDNA) preventing efficient conversion of unmethylated cytosines. Increased dsDNA formation can also be due to an excess concentration of DNA in the reaction which interferes with the ratio of bisulfite to DNA and also increases the pH^29^. Therefore, if the loop structure of mtDNA is not opened completely, it will be difficult to achieve complete denaturation and form single strand. As a result, the bisulfite conversion efficiency will be reduced. All in together, no matter what sample types or which kind of DNA extractions we used for detect mtDNA methylation, it is best to fragment DNA by using restriction endonucleases that will not cut the sequence of interest to insure the bisulfite conversion efficiency.

### Human mitochondrial genome DNA methylation profiling

**Figure 3:**
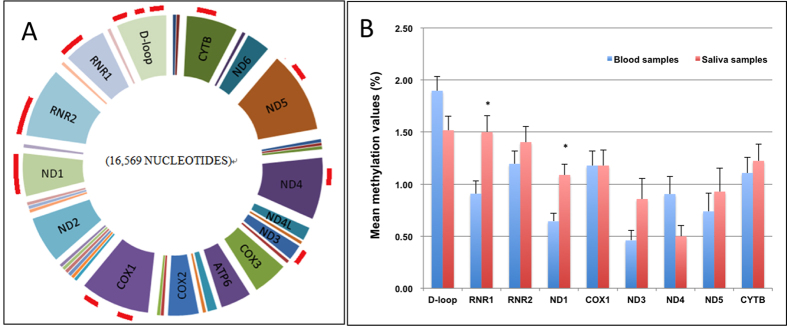
Mean methylation values of 9 regions in human mtDNA. (**A**) The distribution of regions we detected on human mtDNA were marked as red line outside the mtDNA circle. (**B**) Mean methylation values of 9 regions in human mtDNA detected by bisulfite pyrosequencing. Data are represented as mean ± SE. ^*^P < 0.01 *vs* blood samples.

Applying the developed methods of BamHI treatment and bisulfite pyrosequencing, we detected mitochondrial genome methylation of blood samples and saliva samples from 14 unrelated individuals. This detection system covers most part of mtDNA including D-loop region, 12 S rRNA, 16 S rRNA, ND1, COXI, ND3, ND4, ND5, CYTB, which can measure 83 CpG sites including 16 CpG sites in the D-loop region, 21 CpG sites in two rRNA gene regions, and 46 CpG sites in six protein coding regions. The results showed that, regardless of sample types, other than two CpG sites within D-loop region, the methylation levels of the rest of the 81 CpG sites were all less than 3%. The mean methylation levels at CpG16 site (nt544) and CpG429 site (nt16411) within D-loop region were relatively high and obviously prominent, which were both about 5% in blood samples and saliva samples. (Supplementary Table 3). The mean methylation values of nine regions were all below 2% (Fig. 3). Other than 12 S rRNA (*P* = 0.007) and ND1 (*P* = 0.001), there were no significant differences between different tissues at most detected regions (Fig. 3).

Among the nine detected regions, some of them have never been reported, and it is the first time that methylation status has been obtained for ND1, COX1, ND3, ND4, ND5, and CYTB. However, we cannot rule out the possibility that other CpG sites we didn’t analyze may be methylated at high level. But at least, COII and ATP6 have been reported to be unmethylated by Hong *et al*.^20^. Because bisulfite pyrosequencing cannot measure the methylation of non-CpG sites, we also cannot exclude the possibility that there exists methylation outside CpG in mtDNA. Bellizzi *et al*.^15^ has reported that mtDNA methylation of mouse and human blood or cultured cells particularly occurred within non-CpG nucleotides. However, Hong *et al*.^20^ analyzed published genome-wide bisulfite sequencing data sets and the results indicated the lack of cytosine methylation in mtDNA either at CpG site or non-CpG sites, which was also be confirmed by next-generation sequencing. Several studies documented cytosine methylation in mtDNA by using affinity-based methods such as immunoprecipitation^14^ or ELISA^12^,^13^, did not provide any information on the sites or frequency of CpG methylation of the targets. Our data provided the accurate methylation values at each detected CpG site across most regions of human mtDNA. Except two independent CpG sites in D-loop region, the methylation degree of the remaining 81 CpG sites are all less than 3%. The mean methylation levels at CpG16 site (nt544) and CpG429 site (nt16411) within D-loop region were relatively high and obviously prominent, which were both about 5% in blood samples and saliva samples. It has been reported that the interactions of mitochondria DNA-methyltransferase1 (mtDNMT1) with mtDNA appear to be CpG dependent and particularly evident in the D-loop region^14^. D-loop region carries the mitochondrial H strand origin of replication (O_H_). Therefore, higher methylation status at this region might be related with the mtDNA replication, which is need further studies to confirm.

In addition, the sensitivity of pyrosequencing technology for analyzing CpG methylation is 5% and the default value of passed quality of bisulfite treatment is 4.5% at non-CpG cytosine. If the methylation value is close to the threshold, we cannot distinguish the methylation signal from incomplete bisulfite conversion signal. Therefore, the methylation value less than 5% is nonsense and can be considered as non-methylated, and only if the methylation value is lager than 5% under passed quality pyrograph, the methylation result is reliable. With respect to our results, for linear mtDNA, the average CpG methylation degree of 9 detected regions were all less than 5%, indicating that there was no CpG methylation at these regions of mtDNA. Furthermore, understanding of the biological impact of DNA methylation requires the critical information of the DNA methylation pattern, including the methylation status of contiguous sites^20^. With regard to the whole regions, the mean methylation values of nine regions are all less than 2%. Therefore it seems that such low level of mtDNA methylation would have limited or absent functional significance in the control of mitochondrial gene expression, as previously suggested by Shmookler^9^.

Why is DNA methylation in mtDNA very low or even absent? In vertebrate nDNA, a methyl group is added to the 5′ position of the base cytosine to generate 5 mC by the action of DNMT. In this process, S-adenosylmethionine (SAM) is required as a methyl donor. Recently some studies reported that mitochondria contain the machinery required to epigenetically modify mtDNA^11^,^14^,^17^,^30^,^31^,^32^,^33^. However there is some discordancy with regard to these results. Shock *et al*.^14^ identified the mitochondrial targeting sequence for DNMT1 in mouse and human, which drives the translocation of mtDNMT1 to the mitochondria, but did not observe the presence of DNMT3a or DNMT3b in mouse and human cells. However, a study by Wong *et al*.^17^ demonstrated the presence of DNMT3a in mitochondria of adult mouse CNS, skeletal muscle, and testes and human cerebral cortex, while DNMT1 was not detected in adult mouse CNS or skeletal muscle mitochondria. Although it has been reported that cytosolic SAM can be transported into mitochondria by the SAM carrier (SAMC), the exact role of SAM into mitochondria remains poorly understood^11^,^33^. Therefore, whether there is methylation machinery in mitochondria still needs to be reevaluated.

In conclusion, we are the first to show that the circular structure of mtDNA has a great impact on bisulfite conversion efficiency, and thereby affects subsequent pyrosequencing success rate. Even though the sequencing is passed, the quantitative methylation value is significantly higher than the actual value, leading to overestimation of mtDNA methylation. This effect caused by loop structure occurs in blood samples but not in saliva samples. Using BamHI treatment and bisulfite pyrosequencing, human mtDNA methylation profiling of 83 CpG sites covering nine regions (D-loop, 12 S rRNA, 16 S rRNA, ND1, COI, ND3, ND4, ND5, CYTB) was mapped and the average methylation levels of all nine regions are below 2%, indicating CpG methylation in human mtDNA is a rare event at most regions.

## Materials and Methods

### Samples

According to the principle of informed consent, peripheral blood samples were collected from 154 healthy unrelated individuals, and saliva samples were collected from 24 healthy unrelated individuals. For collection of saliva, the volunteers were asked to rinse mouth with mouthwash and 3 mL saliva was collected to a sterile tube. All the participants signed informed consent. In addition, 7 lung tissues were collected from the autopsies of forensic cases under the consent of their most direct relatives. The death causes of these 7 individuals were all not related with lung diseases. This study passed the ethical review and approved by Hebei Medical University Biomedical Ethics Committee. All of the methods were carried out in accordance with the approved guidelines.

### Bioinformatic analysis and primer design of mitochondrial genome CpG sites

Bioinformatic analysis of mitochondrial genome infers a total of 435 CpG sites unevenly distributed across the whole mtDNA, with the highest CpG density in the light chain replication origin (O_L_) (5721~5798 bp), and the minimum density in tRNA genes (Supplementary Table 1). Twenty pairs of primers for amplifying specific mtDNA segments and the corresponding sequencing primers were designed using Assay Design Software. These 20 specific products named MT1~MT20 cover both D-loop region and coding region involving 83 CpG sites. The basic information of 20 sequences is shown in Table 1, and the primer sequences are shown in Supplementary Table 2.

### DNA extraction and quantification

Whole genomic DNA (including mtDNA) was extracted from blood samples and lung tissues using QIAamp DNA Mini and Blood Mini kit (Qiagen, Germany) according to the manual book. DNA extraction form saliva samples was performed by silica and guanidinium isothiocyanate method (Taizhou Stellar Biotechnology Services Ltd, China). DNA concentration and purity were analyzed by ND1000 nucleic acid protein quantitative analyzer. The average concentration of DNA in blood samples and saliva samples were 171 ng/μl and 94 ng/μl, and the average OD260/280 for both samples were 1.90, 1.76 respectively. DNA integrity was verified by 1% agarose gel electrophoresis using randomly selected 60 blood samples and all of the saliva samples.

### BamHI treatment

BamHI specifically recognizes GGATCC and cuts at the position of G/G. There is only one BamHI restriction site in mtDNA at nt14258~nt14263. So after BamHI treatment, the loop of mtDNA can be opened and form a complete linear DNA. DNA was incubated with BamHI at 37 °C for 4 hours in a 20 μl reaction system comprising DNA 1 μg, 10× Buffer 2 μl, BamHI 2 μl, to get complete cutting.

### Bisulfite conversion, PCR and pyrosequencing

Bisulfite conversion was performed using the EZ DNA Methylation-Gold Kit (Zymo Research, USA) according to the manufacturer’s protocol. The converted DNA was amplified to get specific mtDNA fragments using specific primers (Supplementary Table 2) and GoTaq Green Master Mix (Promega, USA). The total reaction volume was 50 μl containing GoTaq Green Master Mix 25 μl, nuclease free water 22 μl, 10 μM sense primer and anti-sense primer 1 μl, and converted genomic DNA 30 ng. The cycle conditions were an initial denaturation at 95 °C for 5 min, 48 cycles of 95 °C for 15 s, 51~57 °C for 30 s, 72 °C for 15 s, and a final extension at 72 °C for 5 min. PCR products were subjected to gel electrophoresis on 2% agarose. If we obtained a specific fragment, then the PCR products could be analyzed by pyrosequencing as follows.

The single strand of PCR products coupled with biotin was purified by mixing 37 μl PCR products with 40 μl binding buffer and 3 μl avidin beads for 30 min with gentle shaker at room temperature, followed by washing with 70% ethanol for 5 s, denaturing with denaturation buffer for 15 s, and washing with washing buffer for 15 s. The purified single strand was annealed with 1.6 μl sequencing primer and 38.4 μl annealing buffer at 80 °C for 5 min. The sequences of the sequencing primers were shown in Supplementary Table 2. After setting up the inner control for testing the efficiency of bisulfite conversion, the pyrosequncing was conducted using PyroMark ID and the quantitative methylation level of each CpG site were obtained. All of the above methods were carried out in accordance with the approved guidelines.

### SEQUENOM EpiTYPRE MassARRAY

To validate the results of pyrosequencing, the mtDNA methylation status of fourteen blood samples was reanalyzed using SEQUENOM MassARRAY platform (CapitalBio, Beijing, China). PCR primers were designed with Methprimer (http://epidesigner.com). For each reverse primer, an additional T7 promoter tag for in vivo transcription was added, as well as a 10-mer tag on the forward primer to adjust for melting temperature differences. Three pairs of primers were designed and used to amplify 3 fragments of mtDNA, from which 31 CpG sites were detected totally. The detailed information was listed in Supplementary Table 4. The bisulfite converted DNA was amplified using PCR Accesory set (Sequenom, San Diego, CA) and the amplification products were cleaned with shrimp alkaline phosphatase using Mass CLEAVE kit (Sequenom, San Diego, CA) to dephosphorylate all unincorporated nucleotides. After purification *in vitro* transcription of the reverse strand and base-specific (C or T) cleavage by RNase A was performed. The cleavage products, which differ in mass and length were spotted onto a MALDI matrix-containing SpectroCHIP and subjected to MALDI-TOF MS. The mass spectra was collected by MassARRAY Spectrometer and analyzed by EpiTYPER software version 1.0 (Sequenom, San Diego, CA).

### Statistical analysis

Statistical analyses were performed using the SPSS13.0 statistical software. Paired *t* test, variance analysis, Wilcoxon test were adopted, and a two-sided *P* < 0.05 was considered statistically significant.

## Additional Information

**How to cite this article**: Liu, B. *et al*. CpG methylation patterns of human mitochondrial DNA. *Sci. Rep.* **6**, 23421; doi: 10.1038/srep23421 (2016).

## Supplementary Material

Supplementary Information

## Figures and Tables

**Table 1:** Brief information of mtDNA sequences detected by bisulfite pyrosequencing.

No. of sequence	Domain	Product	Location	CpG Location	CpG number
MT1	D-Loop	–	8–15	3–8	6
MT2	D-Loop	–	463–609	14–16	3
MT3	rRNA	s-rRNA(12 S rRNA)	1182–1398	37–39	3
MT4	rRNA	s-rRNA(12 S rRNA)	1358–1711	42–45	4
MT5	rRNA	l-rRNA(16 S rRNA)	2495–2626	64–67	4
MT6	rRNA	l-rRNA(16 S rRNA)	2767–2915	72–76	5
MT7	rRNA	l-rRNA(16 S rRNA)	2879–3170	79–83	5
MT8	protein	NADH dehydrogenase subunit 1	3324–3520	96–100	5
MT9	protein	NADH dehydrogenase subunit 1	3469–3596	102–104	3
MT10	Protein	NADH dehydrogenase subunit 1	3596–3798	106–109	4
MT11	protein	NADH dehydrogenase subunit 1	3832–4090	114–118	5
MT12	protein	cytochrome oxidase subunit I	6077–6235	169–172	4
MT13	Protein	cytochrome oxidase subunit I	6920–7107	196–199	4
MT14	protein	NADH dehydrogenase subunit 3	10088–10307	287–293	7
MT15	protein	NADH dehydrogenase subunit 4	11712–11837	325–327	3
MT16	protein	NADH dehydrogenase subunit 5	13878–14045	373–375	3
MT17	protein	cytochrome b	15443–15652	414–418	5
MT18	protein	cytochrome b	15648–15800	419–421	3
MT19	D-loop	–	16045–16222	424–426	3
MT20	D-loop	–	16368–16490	429–432	4

**Table 2:** Comparison of the failure reason of pyrosequencing between BamHI treated group and non-BamHI treated group.

Failure reason	Non open-loop group				Open-loop group			
	MT1	MT3	MT10	MT13	MT1	MT3	MT10	MT13
Blood								
Failed bisulfite conversion	100%(16/16)	10%(1/10)	81.82%(27/33)	89.19%(33/37)	0	50%(2/4)	0	6.67%(1/15)
Lack of data	0	10%(1/10)	0	0	28%(7/25)	0	33.33%(1/3)	13.33%(2/15)
Possible dispensation error/Failed reference sequence pattern	0	0	0	2.70%(1/37)	72%(18/25)	0	0	0
Low signal-to-noise ratio	0	80%(8/10)	18.18%(6/33)	8.11%(3/37)	0	50%(2/4)	66.67%(2/3)	80%(12/15)
Saliva								
Failed bisulfite conversion	0	0	0	0	0	66.67%(2/3)	0	0
Lack of data	50%(1/2)	0	0	0	100(3/3)	0	0	0
Possible dispensation error/Failed reference sequence pattern	0	0	0	0	0	33.33%(1/3)	0	0
Low signal-to-noise ratio	50%(1/2)	100%(2/2)	100%(2/2)	100%(1/1)	0	0	100%(1/1)	100%(2/2)

**Table 3:** *D* values of methylation levels of the DNA same sample with or without BamHI treatment detected by bisulfite pyrosequencing.

CpG location	Blood samples			Saliva samples		
	n	mean ± S/M(QR)	*P*value	n	mean ± S/M(QR)	*P*value
CpG3	32	7.00(2.00)	**<0.001**	15	0.00(0.00)	0.317
CpG4	32	20.19 ± 4.64	**<0.001**	15	−0.63 ± 1.47	0.117
CpG5	32	11.00(4.00)	**<0.001**	15	0.00(0.50)	0.246
CpG6	32	16.22 ± 3.56	**<0.001**	15	0.43 ± 2.33	0.483
CpG7	32	10.00(4.00)	**<0.001**	15	0.20 ± 2.30	0.741
CpG8	32	4.00(1.75)	**<0.001**	15	2.37 ± 1.76	**<0.001**
mean	32	11.50(3.00)	**<0.001**	15	0.43 ± 1.15	0.166
CpG37	24	2.00(3.75)	**<0.001**	14	−1.00(6.00)	0.072
CpG38	24	1.50(2.00)	**0.001**	14	0.60 ± 2.10	0.287
CpG39	24	0.00(2.00)	0.721	14	1.47 ± 2.03	**0.014**
mean	24	1.50 ± 1.38	**<0.001**	14	0.47 ± 1.64	0.290
CpG106	23	6.00(2.00)	**<0.001**	14	0.00(2.00)	0.087
CpG107	23	9.00(3.00)	**<0.001**	14	−0.20 ± 1.31	0.563
CpG108	23	5.00(2.00)	**<0.001**	14	0.00(1.00)	0.285
CpG109	23	3.00(2.00)	**<0.001**	14	0.00(1.00)	**0.041**
mean	23	6.00(2.00)	**<0.001**	14	0.00(0.50)	0.174
CpG196	13	9.08 ± 2.47	**<0.001**	14	0.00(0.00)	0.414
CpG197	13	7.00(3.00)	**0.001**	14	0.07 ± 1.59	0.873
CpG198	13	7.00(1.00)	**0.001**	14	0.00(2.00)	0.201
CpG199	13	6.00(1.00)	**0.001**	14	0.13 ± 1.36	0.709
mean	13	7.00(1.50)	**0.001**	14	0.20 ± 1.15	0.510

*D* values equal to methylation value with BamHI treatment minus without BamHI treatment. *D* values are represented with mean ± S or M (QR) when *D* values do not meet normalized distribution.

**Table 4:** Comparison of methylation levels of the same blood DNA sample with or without BamHI treatment detected by Sequenom MassARRAY.

Amplicon name	CpG location		Methylation level (%)		*P*value
		N	Without BamHI treatment	With BamHI treatment	
Sequenom_1	CpG360	14	12.57 ± 1.60	2.29 ± 0.24	**0.001**
	CpG361	14	12.57 ± 1.60	2.29 ± 0.24	**0.001**
	CpG362	14	6.57 ± 0.23	1.64 ± 0.25	** < 0.001**
	CpG364	14	4.36 ± 0.32	1.43 ± 0.31	** < 0.001**
	CpG365	14	1.21 ± 0.47	0.57 ± 0.23	0.104
	CpG367	14	5.29 ± 0.29	1.50 ± 0.27	** < 0.001**
	CpG368	14	3.43 ± 0.31	1.29 ± 0.27	** < 0.001**
	CpG369	14	4.43 ± 0.23	1.00 ± 0.30	** < 0.001**
	CpG370	14	7.29 ± 0.34	1.86 ± 0.27	** < 0.001**
	CpG371	14	4.36 ± 0.29	1.29 ± 0.35	**0.001**
Sequenom_2	CpG225	14	27.79 ± 3.32	6.86 ± 0.84	**0.001**
	CpG226	14	5.43 ± 0.17	2.92 ± 0.35	**0.003**
	CpG227	14	12.29 ± 0.82	4.21 ± 0.44	** < 0.001**
	CpG228	14	12.29 ± 0.82	4.21 ± 0.45	** < 0.001**
	CpG229	14	2.79 ± 0.21	2.00 ± 0.18	**0.026**
	CpG230	14	26.21 ± 6.48	5.50 ± 0.78	**0.001**
	CpG231	14	11.57 ± 0.84	4.07 ± 0.46	**0.001**
	CpG232	14	11.57 ± 0.84	4.07 ± 0.59	** < 0.001**
	CpG233	14	14.14 ± 0.80	3.50 ± 0.29	** < 0.001**
	CpG235	14	5.14 ± 0.23	3.07 ± 0.46	**0.002**
	CpG236	14	13.86 ± 0.49	4.50 ± 0.31	** < 0.001**
	CpG237	14	13.86 ± 0.49	4.50 ± 0.31	** < 0.001**
	CpG238	14	6.14 ± 0.83	3.57 ± 0.39	**0.005**
	CpG239	14	3.36 ± 0.31	2.00 ± 0.39	**0.007**
	CpG240	14	3.36 ± 0.31	2.00 ± 0.39	**0.007**
	CpG241	14	10.93 ± 1.13	2.50 ± 0.31	**0.002**
	CpG243	14	4.29 ± 0.52	1.86 ± 0.44	**0.005**
Sequenom_3	CpG106	14	5.93 ± 0.56	3.07 ± 0.35	** < 0.001**
	CpG107	14	3.71 ± 0.19	1.14 ± 0.25	** < 0.001**
	CpG108	14	3.71 ± 0.19	1.14 ± 0.25	** < 0.001**
	CpG109	14	3.43 ± 0.92	1.07 ± 0.29	**0.006**

**Table 5:** Comparison of methylation levels of the same lung DNA sample with or without BamHI treatment detected by bisulfite pyrosequencing.

Amplicon name	CpG location	N	Methylation level (%)		*P*value
			Without BamHI treatment	With BamHI treatment	
MT1	CpG3	7	2.86 ± 0.59	1.57 ± 0.75	0.318
	CpG4	7	5.86 ± 1.06	1.43 ± 0.95	**0.017**
	CpG5	7	5.57 ± 1.67	1.14 ± 0.74	**0.026**
	CpG6	7	2.57 ± 1.34	1.29 ± 0.84	0.620
	CpG7	7	4.29 ± 0.78	2.29 ± 1.13	0.073
	CpG8	7	0.57 ± 0.57	2.29 ± 1.11	0.318
	Mean	7	4.00 ± 0.95	1.57 ± 0.20	**0.040**
